# Expression and prognostic significance of cox-2 and p-53 in hodgkin lymphomas: a retrospective study

**DOI:** 10.1186/1746-1596-5-19

**Published:** 2010-03-26

**Authors:** Nagehan O Barisik, Suheyla Bozkurt, Mahmut Gumus, Isik Kaygusuz, Nimet Karadayi, Emine Bas, Mahmut Bayik, Tulay Tecimer

**Affiliations:** 1 Kartal Research And Education Hospital Pathology Department/Istanbul-Turkey; 2 Marmara University Pathology Department/Istanbul-Turkey; 3 Kartal Research And Education Hospital Oncology Department/Istanbul-Turkey; 4 Marmara University Hematology Department/Istanbul-Turkey

## Abstract

**Background:**

Cyclooxygenase (cox) is the rate-limiting enzyme, which catalyzes the conversion of arachidonic acid into prostaglandins and contributes to the inflammatory process. Cyclooxygenase-2 (cox-2), which is one of the two isoforms, plays a role in tumor progression and carcinogenesis. p53 contributes to apoptosis, DNA renewal and cell cycle. Studies concerning the relationship of cox-2 and p53 expressions and carcinogenesis are available, but the association between cox-2 and p53 in Hodgkin lymphoma (HL) is not exactly known.

In our study, we examined the association of cox-2 and p53 expression, with age, stage, histopathological subtype, and survival in HL. We also examined correlation between cox-2 and p53 expression.

**Methods:**

Cox-2 and p53 expressions in Hodgkin-Reed Sternberg cells (HRS) were examined in 54 patients with HL depending on cox-2 expression, stained cases were classified as positive, and unstained cases as negative. Nuclear staining of HRS cells with p53 was evaluated as positive. The classifications of positivity were as follows: negative if<10%; (1+) if 10-25%; (2+) if 25-50%; (3+) if 50-75%, (4+) if >75%.

**Results:**

Cox-2 and p53 expressions were found in 49 (80%) and 29 (46%) patients, respectively. There were differences between histological subtypes according to cox-2 expression (p = 0.012). Mixed cellular (MC) and nodular sclerosing (NS) subtypes were seen most of the patients and cox-2 expression was evaluated mostly in the mixed cellular subtype.

There were no statistically significant relationships between p53 and the histopathological subtypes; or between p53, cox-2 and the factors including stage, age and survival; or between p53 and cox-2 expression (p > 0.05).

**Conclusion:**

Considering the significant relationship between the cox-2 expression and the subtypes of HL, cox-2 expression is higher in MC and NS subtypes. However the difference between these two subtypes was not significant. This submission must be advocated by studies with large series

## Introduction

Hodgkin lymphomas (HL) are malignancies derived from neoplastic Reed Sternberg (RS) cells which are found in the inflammatory media consist of plasma cells, eosinophils and histiocytes. The RS and its variant Hodgkin (H) cells form 1-3% of the whole mass [1]. It generates about 1% of the overall cancers and 30% of the lymphoid malignancies [1,2]. In the polymerase chain reaction (PCR) analyses performed to determine the function and histogenesis of Hodgkin-Reed Sternberg (HRS) cells, it is estimated that these cells are originated from the germinal center B cells and they have somatically mutated immunoglobulin (Ig) gene rearrangements. Not only the Ig expression levels of the HRS cells which are identified as B cells, but also their levels of transcription that is mediated by inactivation of Ig promoters, are disturbed. It is reported that HRS cells can block the apoptosis through an apoptotic mechanism which is not clearly understood, when the normal B cells go into apoptosis after loosing their capacity of Ig expression. It is suggested in the literature that Ebstein-Barr virus (EBV) infection and the nuclear factor kappa B (NFkB) released from HRS cells also act through this way [1,3-6].

Although various factors for the etiology have been widely studied, it is estimated that EBV infection contributes in some of the cases [2,3]. It is reported in large series of epidemiologic studies that the risk of HL is lower in people using anti-inflammatory drugs, compared to the ones who do not use or who irregularly use nonsteroidal anti-inflammatory drugs (NSAIDs) [3].

The best known target of NSAIDs is the cyclooxygenase (cox) enzyme. Cox enzyme catalyzes the synthesis of prostaglandins (PGs) from arachidonic acid. Two isoforms of the cox gene have been identified. Cyclooxygenase-1(cox-1) is constitutively expressed on cell membranes in normal tissues and related to physiological functions, such as cytoprotection of the stomach and control of platelet aggregation: and cyclooxygenase-2 (cox-2) is cytoplasmic in location, not detectable in most of the normal tissues and induced by inflammatory and mitogenic stimuli. Furthermore expression of cox-2 is stimulated by oncogenes, growth factors, cytokines, tumor promoters and it has been shown to inhibit apoptosis [7,8]. In several tumors such as colon, esophagus, stomach, cervix and breast, cox-2 is found to be associated with carcinogenesis and tumor progression [7,9-14].

The p53 gene located on the short arm of chromosome 17 has been described as a tumor suppressor gene producing a 53 kD nuclear binding protein [15,16]. It is thought that p53 has a role in regulation of normal cell cycle, apoptosis and in response to DNA damage [16]. It leads to selective growth and tumor formation by the replication of mutant p53 and deregulating cell cycle [17]. It is reported that mutant p53 acts by changing the genetic structure in lung, breast, pancreas and lymphoid malignancies [5,16,17]. It is declared in the previous studies that there might be p53 mutations and the antiapoptotic effect of p53, in the HRS cells [5,18,19].

The aim of our study is to determine the association between cox-2 and p53 expressions with the histological subtypes, age, survival and tumor dissemination, in HL cases.

## Materials and methods

In this study, a total of 54 patients were enrolled who were diagnosed with HL in Dr. Lutfi Kirdar Kartal Education and Research Hospital and University of Marmara Medical School between 2000 and 2007.

The cases were classified according to age, survival, stage and the histological subtypes.

Ann Arbor staging system was performed for classification, and staging results were obtained from the patients' files. All samples were fixed in formalin and embedded in paraffin. The histological slides of all of the patients were reviewed by two blinded pathologists and categorized according to the WHO classification of hematological malignancies into nodular lymphocyte predominant (NLP), nodular sclerosing (NS), lymphocyte-rich (LR), mixed cellular (MC) and lymphocyte depleted (LD) type [1]. After preparing the slides form the appropriate paraffin block, immunohistochemical assessment was performed for cox-2 and p53, for diagnostic evaluation.

### Immunohistochemistry

Three μ thick sections were placed on 3-aminopropylethylene-covered slides and deparaffinized at 60°C for 1 hour. The slides were dipped in xylol for 15 minutes for deparaffinization, soaked with alcohols for 15 minutes and washed in distilled water. Afterwards, they were applied in pH 6 citrate buffer for antigen retrieval process in microwave oven for 20 minutes. Endogenous peroxidase activity was blocked by 10 minutes of incubation with 3% hidrogen peroxidase. To prevent nonspecific bindings, blockage was performed for 10 minutes (Dako Cytomation protein block serum-free, ref × 0909, lot 10016138, Denmark). Cox-2 was incubated with monoclonal mouse anti-human (Dako Cytomation EnVision Clone CX294, Code M3617, 1:100 dilution for 90 min, Denmark). They were incubated for 30 minutes with polyvalent HRP polymer (Spring Bioscience cat: DPE-125, Lot: 70918, California). P53 was incubated with monoclonal mouse anti-human (Dako Cytomatin En Vision Clone DO-7, Code N1581 ready-to-use for 30 min, Denmark)

AEC chromogen system was applied to slides for 15 minutes (Spring Bioscience catalog: ASS-125 ready to use, California). Contrast staining was done with mayer-hematoxylene and slides were covered with water-based cover material.

### Evaluation of immunostaining

The cases, in which cytoplasmic cox-2 expressions were determined in their HRS cells, were accepted as positive. RS cells are large with variable amount of cytoplasm that is eosinophilic or amphophilic. The nuclei are large thick nuclear membranes. Also there can be two nuclei, symmetric with ''mirror image" appearance. On the other hand H cells mono nuclei or multinuclei and these cells have positive staining with CD15 or and CD30 antibodies in immunohistochemical staining. Nuclear staining of p53 was evaluated as follows: negative if <10%; (1+) if 10-25%; (2+) if 25-50%; (3+) if 50-75%; (4+) if >75% of HRS cells were positive [7].

### Statistical analysis

Statistical analyzes were performed using SPSS 13.0. Chi-square test was used in the comparison of classified data as well as in evaluation of descriptive statistical methods (median range). Survival analyses were performed using the Kaplan-Meier method. Results were evaluated in 95% confidence interval and p < 0.05 was determined as the level of significance.

## Results

### Patients and follow-up

This study included 54 patients: the mean age at diagnosis was 37 years. In accordance with the histological subtypes, there were 26 (48.1%) cases of NS subtype, 24 (44.4%) cases of MC subtype, 3 (5.6%) cases of LR subtype, and 1 (1.9%) case of NLPHL. Data from clinical follow-up were obtained in 23 patients, up to 66 months. The mean follow-up was 39 (5-66) months.

Among 23 patients, there were 20 (90%) cases administered with ABVD, 1 (4.5%) case with ABVD+CMOPP and 1 (4.5%) case with ABVD+CBVP-K. Three (13%) cases were in stage I, 6 (26.1%) cases were in stage II, 10 (43.5%) cases were in stage III and 4 (17.4%) cases were in stage IV. 9 (39%) cases were administered with RT but 14 (60.9%) cases were not (table 1).

**Table 1:** Distrubution of Parameters

		N	%
**Age (n = 54)**	**≤ 35 years**	24	44,4
	**>35 years**	30	55,6

**Histological type (n = 54)**	**NS**	26	48,1
	**MC**	24	44,4
	**LR**	3	5,6
	**NLP**	1	1,9

**Therapy (n = 22)**	**ABVD**	20	91,0
	**ABVD+CMOPP**	1	4,5
	**ABVD+ CBVP-K**	1	4,5

**Stage (n = 23)**	**1**	3	13,0
	**2**	6	26,1
	**3**	10	43,5
	**4**	4	17,4

**Bulky (n = 23)**	**Yes**	2	8,7
	**No**	21	91,3

**RT (n = 23)**	**Yes**	9	39,1
	**No**	14	60,9

### Cox-2 and p53 expression

**Figure 1:**
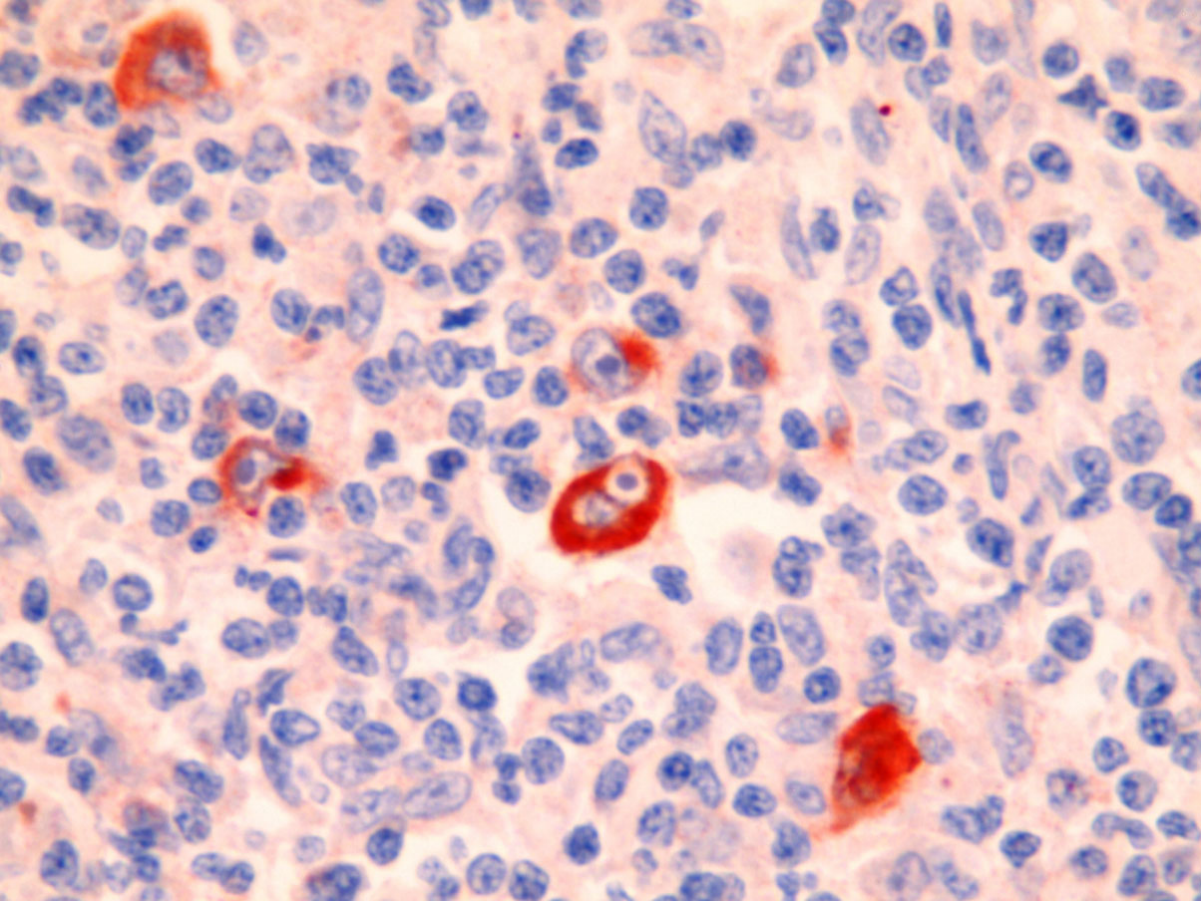
**(cox-2 × 100) cox-2 exspresion in H/R-S cells**.

**Figure 2:**
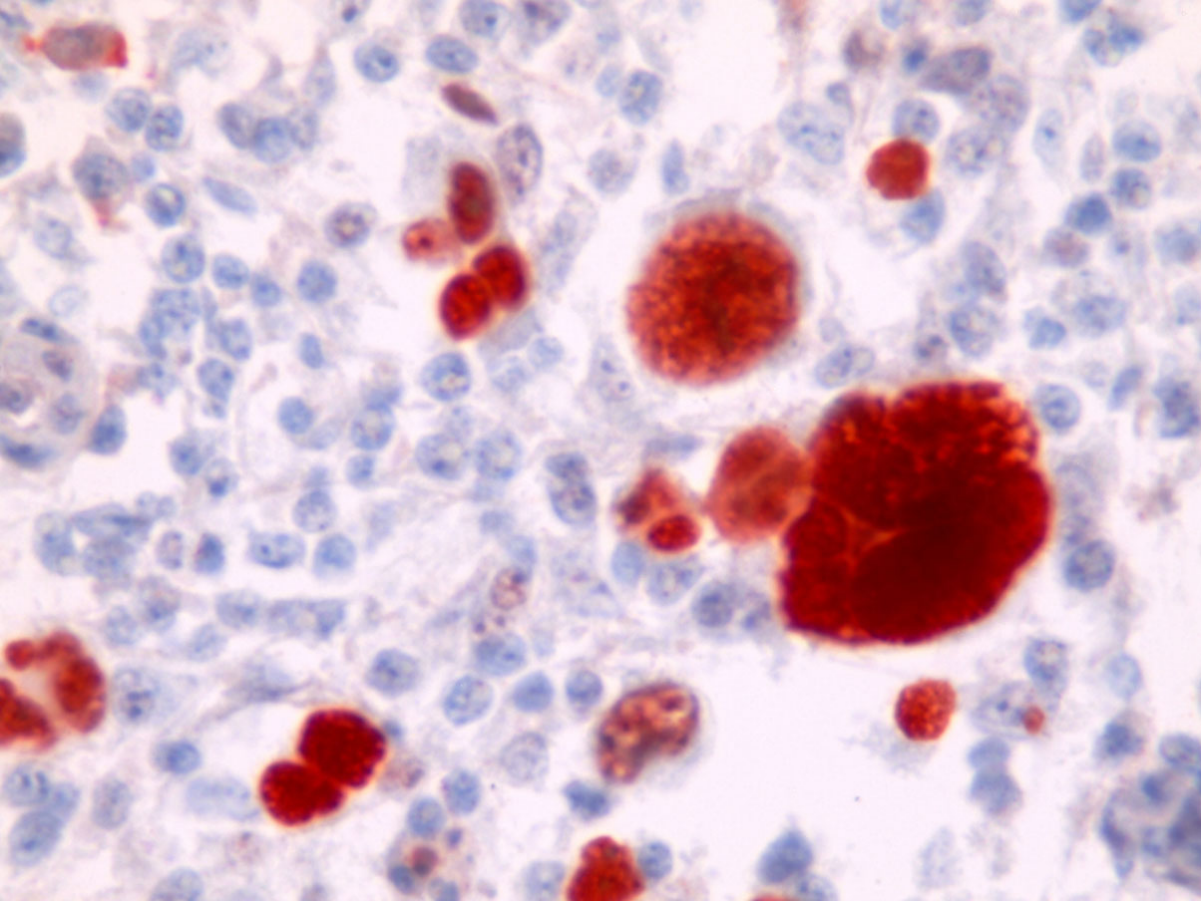
**(p53 × 100) p53 expression in H/R-S cells**.

Immunoreactivity with Cox-2 was seen in 49 (80%) of the 54 cases with HL (figure 1). On the other hand p53 staining was seen in 26 (48%) of the 54 cases (figure 2). Significant relationship was determined between the cox-2 expression and the histological subtypes (p = 0.012). Considering the NS and MC subtypes, which generate the majority of the cases, MC subtype demonstrated more staining than NS subtype. However there is no statistically significant difference between NS and MC subtypes considering cox-2 expression (p = 0.61). All cases were stained with cox-2 in lymphocyte rich type which generates the minority of the cases. However no staining was detected in NLP type. No statistically significant results were derived from the evaluation of cox-2 expression according to their stages in 23 cases who had complete follow-up (p = 0.142). Evaluating the cox-2 expression in terms of age, 24 cases were <35 and 30 patients were >35 years old. There was no statistically significant relationship between the cox-2 expression and age (p = 0.367). The association of cox-2 expression with the histological subtypes, stage and age are presented on table 2. No significant relationship was detected between p53 and the histological subtypes, stage and age (p > 0.05) (table 3).

**Table 2:** The association of Cox-2 expression with the histological subtypes, stage and age

	COX-2 expression (negative) n%	COX-2 expression (positive) n%	p value
**Histologic type**			0.012*
LR	0(100)	3(100)	
MC	1(4.2)	23(95.8)	
NS	3(11.6)	23(88.4)	
NLP	1(100)	0(0)	
**Stage**			0.142
Stage I-II	2(22.2)	7(77.8)	
StageIII-IV	0(0)	14(100)	
**Age**			0.367
<35	1	23	
>35	4	26	

* Chi-square test **p < 0.05**

**Table 3:** Histological type, stage, age and p 53 expression

	P53 expression (negative) n%	P53 expression (positive)n %	p value
**Histologic type**			0.078
LR	3(100)	0(0)	
MC	15(62.5)	9(37.5)	
NS	10(38.5)	16(61.5)	
NLP	0(0)	1(100)	
**Stage**			0.669
StageI-II	3(33.3)	6(66.7)	
StageIII-IV	7(50)	7(50)	
**Age**			0.548
<35	11(45.7)	13(44.3)	
>35	17(56.6)	13(53.4)	

^+ ^Chi-square test **p < 0.05**

### The association between cox-2 and p53 expression

The association between cox-2 expression and p53 is evaluated, but no statistically significant relationship was detected (p > 0.05) (table 4).

**Table 4:** The association between cox-2 and p53 expression

P 53 expression	COX-2 expression (negative) n%	COX-2 expression (positive) n%	P value
Negative	2(7.1)	26(92.9)	0.663
Positive	3(11.5)	23(88.5)	
Total	5	49	

^+ ^Chi-square test **p > 0.05**

### The association between cox-2 expression and survival

**Figure 3:**
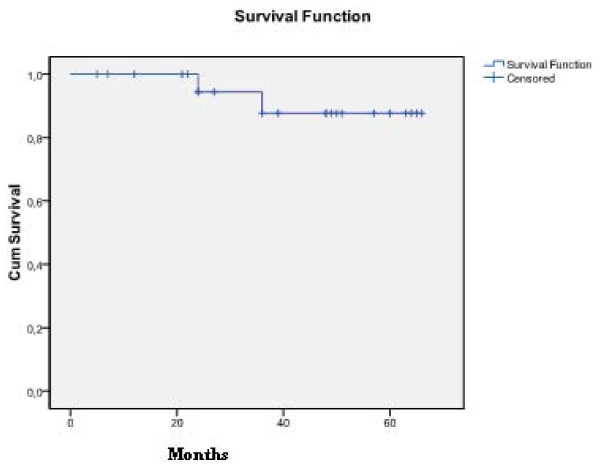
**Association between Cox-2 expression and survival**.

Five years survival was 87.7%, in the follow-up group. The median overall survival could not be achieved (figure 3).

The effects of p53 and cox-2 expressions on survival have been investigated as well, but statistically significant results were not obtained, due to the small number of the potentially follow-up group and death of only two of the patients.

## Discussion

It is known that cox-2 acts in tumor mechanisms including tumor proliferation, differentiation, immunosupression, angiogenesis and inhibition of apoptosis, by synthesis of PGs [20]. In the previous studies it has been demonstrated that cox-2 expression increases in colon, stomach, esophagus, lung, over, cervix, liver, pancreas adenocarcinomas and brain tumors and it is reported as a negative prognostic parameter [12,13,21-24]. Moreover, it is reported in epidemiologic and experimental studies that cox-2 inhibitors and NSAIDs prevent the carcinogenesis [9-11,13,14,20,21,25,26]. A great number of studies regarding the solid organ tumors are available, however, there are very little numbers of studies presented concerning the hematological malignancies in association with the cox-2 expression, and thus the relationship between the lymphoid malignancies and the cox-2 expression is not clearly understood.

Ohsava et al., Hsu et al., and Hazar et al. observed the cox-2 expressions in classical Hodgkin lymphoma (CHL) and detected cytoplasmic cox-2 expression in 7(70%) of 10 CHL cases, 7(70%) of 10 CHL cases 15 (48%) of 31 CHL cases, respectively [7,27]. In our study, the cox-2 expression was determined in 49 (80%) patients among the 54 HL cases.

It is emphasized that cox-2 increases the expression of angiogenic factors such as PGE2, as well as the expression of proangiogenic factors; and also that PGE2 is a strong vascular endothelial growth factor (VEGF) inducer and consequently cox-2 contributes to angiogenesis and increases the tumor proliferation [7,13,22].

The study of Ohsawa et al. on this point investigated the cox-2 expression in association with proliferation index and microvessel density, and demonstrated that Ki67 proliferation index and the microvessel density were 2 and 4 folds greater in cox-2 expressing group [7]. It is known that interleukin-1 (IL-1), interleukin-5 (IL-5), NFkB, granulocyte colony stimulating factor, tumor necrosis factor and many other cytokines are released from the HRS cells (1,28). These cytokines result in B symptoms such as fever, sweating and weight loss which are included within the negative prognostic parameters [1]. Hazar et al. evaluated the association of cox-2 with histological type, age, stage and the response to the therapy. They obtained a lesser response to the therapy in non-HL cases with cox-2 expression and reported cox-2 expression as a negative prognostic parameter [27]. In our study, the association of cox-2 with histological subtypes was investigated and a greater staining was determined in the MC subtype which generates the majority among the overall groups. Since the cytokines are known to induce the cox-2 expression, we can accept that MC type (except lymphocyte poor type) involves more HRS cells, and therefore releases more cytokines compared to the other types. However there is no statistically significant difference between NS and MC subtypes considering cox-2 expression (p = 0.61). With regard to the fact that negative prognostic features such as advanced stage and contiguity dissemination are more often observed in the MC type. In our study, the association of age and survival with cox-2 expression was observed, but no statistically significant relationships were obtained however higher level of cox-2 expression in MC type was noteworthy.

It is suggested that p53 plays an important role in cell-cycle control, maintenance of genomic stability, cell differentiation and programmed cell-death. The mutation or depletion of p53 oncogen is one of the most frequently reported lesions in various types of cancers [4,7,15,16,28,29].

Generally it is accepted that, most of the mutations of the p53 gene result in production of a mutant p53 protein. The half time of the mutant p53 protein is extended; therefore the cells tend to accumulate.

Reports about the abnormal accumulation of p53 in HRS cells are available [2,4,19]. Moreover, the p53 gene mutations may lead to the disturbance of p53 mediated transactivation and this evidence may considered to be the key factor of apoptosis resistance of the p53 gene mutation. However, the association between the high levels of the p53 protein and p53 mutations could not be determined in some of the studies [4]. In a study carried out by Rongen et al., the p53 gene was evaluated in 8 CHL cases and 6 (80%) of them demonstrated p53 gene mutations in their HRS cells [5].

The p53 expression in HRS cells were assessed and shown in 36 (72%) cases among the 50 CHL patients in the study of Gupta et al., in all of the 67 CHL patients in the study of Magio et al. and in 14 (42%) cases among the 33 CHL patients in the study of Ohsawa et al. [7,16,19]. Wang et al. evaluated the association of p53 with the clinical stage, EBV, bcl-2, retinoblastoma gene, p21, Ki67 and topoisomerase-II-alfa and detected p53 gene in 14 patients among the 62 CHL cases. They have detected p53 expression more frequently in stage I, but in a lesser frequency in the advanced stages such as stage III and IV. However, no significant relationship was found between the clinical stages, histological subtypes and the p53 [4]. In this study it is reported that the rate of p53 expression is small; therefore abnormal p53 protein expression may not be effective in the blockage of apoptosis mediated deaths of HRS cells [4]. In our study, p53 expression was detected in 26 (48%) of the 54 cases, but no association was determined with the histological subtype, the stage, age and survival.

Association of cox-2 and p53 was reported in the studies previously performed. Subbaramaiah et al., compared the cox-2 expression between the wild type (wt) p53, mutant p53 and p53 non-expressing mouse fibroblasts and discovered that cox-2 expression was markedly repressed by wt p53. They thought that this investigation may describe why these levels of cox-2 protein are not seen in normal epithelial cells and contrarily why mutant p53 increases the levels of cox-2 expression in malignant tissues. They reported that the cytokines in mutant p53 expressing cells, oncogenes and growth factors can increase cox-2 induction [18].

There are also studies available investigating the cox-2 and p53 expressions in ovarian adenocarcinoma and in head and neck tumors and a positive association between cox-2 and p53 was reported [17,20].

However, Ohsawa et al. did not determine an association between cox-2 and p53 expression [7]. In our study, the association between p53 and cox-2 is also investigated, but no statistically significant evidence was found (p = 0.663).

Consequently, evaluating the association between the histological subtypes and cox-2 expression, statistically significant association between histological subtypes and cox-2 was determined. However, considering both cox-2 and p53, significant association of cox-2 and p53 with age, stage and survival were not detected. Median follow-up was 36 months, in our study and it was below 5 years of follow-up. The statistically significant association between cox-2 and histological types is promising, nevertheless, for the corroboration of the data and in support of including the cox-2 in prognostic parameters, long term follow-up studies with large series are needed. We think that our study is elucidating in this aspect.

## Competing interests

The authors declare that they have no competing interests.

## Authors' contributions

NOB participated in conception and design. SB performed immunohistochemical staining. MG performed the statistical analysis. IK participated in acquisition of data. NK participated in acquisition of data. EB conceived of the study. MB participated in collecting of data. TT participated in writing of the manuscript. All authors read and approved the final version of the manuscript.
